# *Annona cherimola* Miller and Its Flavonoids, an Important Source of Products for the Treatment of Diabetes Mellitus: In Vivo and In Silico Evaluations

**DOI:** 10.3390/ph16050724

**Published:** 2023-05-10

**Authors:** Fernando Calzada, Miguel Valdes, Jesús Martínez-Solís, Claudia Velázquez, Elizabeth Barbosa

**Affiliations:** 1 Unidad de Investigación Médica en Farmacología, UMAE Hospital de Especialidades 2° Piso CORSE, Centro Médico Nacional Siglo XXI, Instituto Mexicano del Seguro Social, Av. Cuauhtémoc 330, Col. Doctores, Mexico City CP 06720, Mexico; 2 Instituto Politécnico Nacional, Sección de Estudios de Posgrado e Investigación, Escuela Superior de Medicina, Plan de San Luis y Salvador Díaz Mirón S/N, Col. Casco de Santo Tomás, Miguel Hidalgo, Mexico City CP 11340, Mexico; jarimarts27@gmail.com (J.M.-S.); rebc78@yahoo.com.mx (E.B.); 3 Área Académica de Farmacia, Instituto de Ciencias de la Salud, Universidad Autonoma del Estado de Hidalgo, Circuito exHacienda La Concepcion s/n, Carretera Pachuca-Atocpan, San Agustin Tlaxiaca CP 42076, Mexico; cvg09@yahoo.com

**Keywords:** *Annona cherimola* Miller, antihyperglycemic activity, flavonoids, in vivo assays, in silico assays

## Abstract

The antihyperglycemic activity of ethanolic extract from *Annona cherimola* Miller (EEAch) and its products were evaluated using in vivo and in silico assays. An α-glucosidase inhibition was evaluated with oral sucrose tolerance tests (OSTT) and molecular docking studies using acarbose as the control. SGLT1 inhibition was evaluated with an oral glucose tolerance test (OGTT) and molecular docking studies using canagliflozin as the control. Among all products tested, EEAc, the aqueous residual fraction (AcRFr), rutin, and myricetin reduced the hyperglycemia in DM2 mice. During the carbohydrate tolerance tests, all the treatments reduced the postprandial peak such as the control drugs. In the molecular docking studies, rutin showed more affinity in inhibiting α-glucosidase enzymes and myricetin in inhibiting the SGLT1 cotransporter, showing ∆G values of −6.03 and −3.32 kcal/mol^−1^, respectively, in α-glucosidase enzymes. In the case of the SGLT1 cotransporter, molecular docking showed ∆G values of 22.82 and −7.89 in rutin and myricetin, respectively. This research sorts in vivo and in silico pharmacological studies regarding the use of *A. cherimola* leaves as a source for the development of new potential antidiabetic agents for T2D control, such as flavonoids rutin and myricetin.

## 1. Introduction

Diabetes Mellitus (DM) type 2 is characterized by polydipsia, polyphagia, polyuria, and weight loss and accounts for 90 to 95% of all diabetes cases [1]. According to the International Federation of Diabetes, there are over 537 million people worldwide living whit in 2021 [2]. Further, the WHO estimates that DM caused 1.6 million deaths in 2016; therefore, it is considered one of the main causes of deaths globally [3]. 

In addition to lack of insulin secretion or resistance in skeletal muscle tissue, this chronic disease has a polygenic origin that includes another factor called ominous octet [4], which leads to an increase in blood glucose levels that is the main characteristic and cause of micro- and macrovascular consequences [5].

Generally, for the therapeutic management of diabetes, drugs with varying chemical structures are used to normalize blood glucose levels through different mechanisms of action [6]. These treatments are administered orally or in the parenteral way for life, which often represents a high financial cost. Moreover, the appearance of adverse effects or hypersensitivity reactions are situations that make it necessary to suspend the treatment or change it constantly [7]. These reasons make the development of new agents with antidiabetic properties important. One of the main targets for the search for new antidiabetic drugs is the inhibition of intestinal glucosidases. Iminosugars synthesized and sugar derivates are potential antidiabetic drugs, showing a favorable impact in glycosidase inhibition, improving their activity through inhibitor synthesis and design of conformational preorganization [8,9]. On the other hand, medicinal plants are an excellent alternative to finding complementary treatments to achieve improved control of hyperglycemia [10].

More than 400 plant species have been registered for the control of diabetes [11]; however, only a few of these have received scientific and chemical evaluations to prove their effectiveness [12]. Compounds in medicinal plants have antidiabetic potential through several mechanisms [13], including the inhibition of glucose uptake in the gut, stimulation of insulin secretion from the pancreas, and many others that can influence its therapeutic potential. 

In this sense, in the Annonaceae family, some genera are characterized by the economic interest of their fruits; such is the case of the genus Annona spp., which consists of many species, of which about 20 are cultivated for said interest [14]. Moreover, several species include compounds that have already been studied for their antidiabetic properties [15]. Among the most cultivated is *Annona cherimola* Miller (*A. cherimola*). It is a perennial tree of edible fruit whose leaves are present almost all year [16]. However, it is known that environmental conditions such as temperature and humidity can modify the growth of its leaves and modify the concentration of the bioactive compounds [17]. *A cherimola* constitutes part of the natural flora in Central America and South America, where it is known as “anona” or “cherimoya” and is widely cultivated and used as a traditional medicinal remedy by local populations to treat various illnesses, including gastrointestinal disorders, worms, and diarrhea [18]. Research has recently intensified mainly due to the discovery of the great potential of the natural products they contain [19]. Further, it is the only species within this genus to which special attention has been paid due to its medicinal and nutritional values, which have been exploited in Europe, where it has been preserved and consumed for the aroma and delicate flavor of its fruits. Hence, its acceptance from the commercial point of view as an exotic fruit is widely disseminated internationally, with a marked interest in its expansion [20]. Its marketing generally goes from the local scale to the international level, and as the cherimoya begins to be better known, it is the object of greater attention by researchers, growers, and consumers from many countries [21]. For this reason, it is necessary to stimulate scientific research related to the biological activity of some of its components, medicinal properties, and its various potentialities in human nutrition. In addition, this species is significant in the conservation and restoration of ecosystems. 

Recent studies have provided information that the leaves of this species have antidepressant [22], pro-apoptotic [23], and antilipidemic activities [24]. Further, previous investigations showed that extract from the leaves of this species has an antidiabetic effect when it is administered orally in acute and subchronic treatment alone [25] or even in combination with the main antidiabetic drugs [26], with a wide frame of security with an LD_50_ over 3000 mg/kg without toxic effects in healthy organs [27]. 

It is known that the leaves of this species contain many kinds of compounds [28] that exert antihyperglycemic activities responsible for their medicinal potential [29], where flavonoids are highlighted due to their wide-ranging properties [30].

Although the antidiabetics [31] and other effects related to the antioxidant mechanisms [32] of the rutin constituent present in a greater quantity in the leaves of this species have already been studied [33], there are no studies that evaluate other components present in lower concentrations in the leaves of *Annona cherimola*.

Thus, the present study aims to assess the effect of the minor components obtained from the leaves of *Annona cherimola* in a diabetic model to show its antihyperglycemic potential that may contribute to validating its use in the treatment of diabetics.

## 2. Results

### 2.1. In Vivo Assays

#### 2.1.1. Acute Evaluation of the Antidiabetic Effect of Ethanolic Extract from *Annona cherimola* and Its Polyphenols

Initially, the acute antihyperglycemic effect of the treatments was measured using a single dose; this was selected to determine the probable activity of the treatments in the short term (2 and 4 h) over the hyperglycemic values. 

The ethanolic extract obtained from the leaves from *Annona cherimola* Miller (EEAch) was determined. It exhibited a significative reduction of hyperglycemia at 2 and 4 h after the administration of 300 mg/kg of EEAch; this effect was significative in comparison with the initial values and comparison with the alloxan-induced type 2 diabetic mice (AIT2D) control (Table 1).

After the fractionation of the extract, we obtained the dichloromethane fraction (DCMFr) and an aqueous residual fraction (AcRFr); both fractions were evaluated to determine their antihyperglycemic effect. When those products were administered, a significant reduction of hyperglycemia was observed at 2 and 4 h in both treatments (Table 1). Being more effective, the AcRFr reduces the hyperglycemic values near normoglycemic mice values.

The purification of AcRFr was carried out using preparative thin-layer chromatography, and this led to the isolation of eight compounds with polyphenolic characteristics, astragalin, chlorogenic acid, hyperin, isoquercitrin, myricetin, narcissin, nicotiflorin, and rutin (see Supplementary Materials). Following the scheme of work, all compounds were evaluated in the AIT2D model to determine which of them reduces hyperglycemia; the effect was compared with the oral antidiabetic drug (OAD) acarbose.

All compounds and acarbose were administered at the dose of 50 mg/kg. We observed that after administration, hyperin, myricetin, nicotiflorin, and rutin significantly reduced the hyperglycemic values at 2 and 4 h, and similar activity was observed in the group treated with acarbose. The effect over hyperglycemic values observed was significant in comparison with the initial values, and in comparison, with the AIT2D control at the respective times of measurement. In the case of isoquercitrin, it significantly reduces hyperglycemic values only at 2 h of the treatment and narcissin at 4 h; both effects were significant in comparison with AIT2D control. On the other hand, astragalin and chlorogenic acid did not generate a significant reduction in hyperglycemia (Table 1). 

The results obtained allowed us to determine that myricetin and rutin were the compounds with the major antidiabetic effect; therefore, both compounds were selected to be evaluated in a subchronic assay as well as EEAc and AcRFr. 

#### 2.1.2. Subchronical Evaluation of the Antidiabetic Effect of Ethanolic Extract from *Annona cherimola*, AcRFr, Myricetin, and Rutin

The products with the best acute antihyperglycemic activity from *A. cherimola* were evaluated in a subchronic assay. 

**Figure 1:**
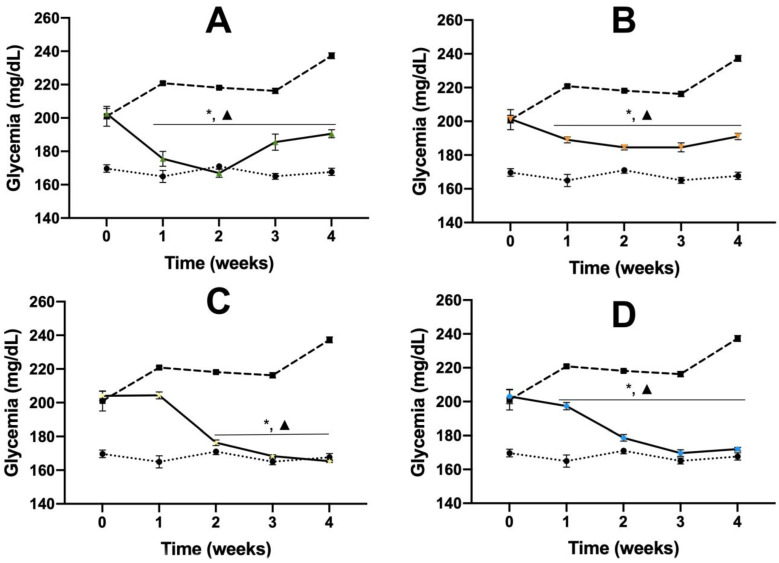

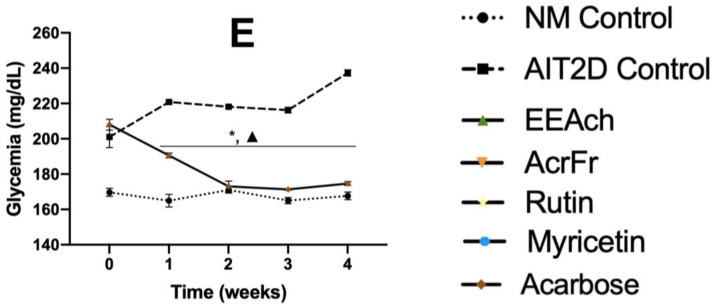
Effect over glycemic values of subchronic administration of the treatments in AIT2D mice. Groups treated with EEAch 300 mg/kg (**A**), AcRFr 300 mg/kg (**B**), rutin 50 mg/kg (**C**), myricetin 50 mg/kg (**D**), and acarbose 50 mg/kg (**E**). Results are expressed as the mean ± SEM, *n* = 6, * *p* < 0.05 vs. initial values; Δ *p* < 0.05 vs. AIT2D control at same week of treatment. NM: normoglycemic mice; AIT2D: alloxan-induced type 2 diabetes mice; EEAch: ethanolic extract of *Annona cherimola* Miller; AcRFr: aqueous residual fraction.

We observed that animals treated with EEAch showed a significant reduction of hyperglycemic values from week one, reaching normoglycemic values in week two; however, in weeks three and four, the glycemic values began to increase again. During the experiment, the hyperglycemia reduction was significant in comparison with the initial values and the AIT2D control (Figure 1A).

The group treated with AcRFr showed a significant reduction of hyperglycemia from week one, this reduction was constant during the experiment, and the antidiabetic effect was significant in comparison with the initial values and the AIT2D control (Figure 1B); however, in this case, the normoglycemic values was never reached. 

In the case of rutin, this treatment shows a significant reduction of hyperglycemia from week two in comparison with the AIT2D control, and reaching normoglycemic values from week three, this effect was observed until the end of the assay (Figure 1C). 

For the other flavonoid, the group treated with myricetin showed an effect similar to the group treated with rutin, i.e., a good reduction of hyperglycemic values from week one. This reduction was significant in comparison with the initial values as well as with the AIT2D control. Moreover, this group reached normoglycemic values from week two, and this activity was observed until the end of the assay (Figure 1D).

Finally, the group treated with acarbose showed a significant reduction from week one; this effect was similar to the observed in the groups treated with the flavonoids rutin and myricetin (Figure 1E). 

The results showed us that rutin and myricetin were compounds with good antihyperglycemic activity in acute and subchronic assays; the next step of our study was to determine their potential inhibitory activity over a-glucosidase enzyme and SGLT-1 cotransporter, two important proteins involved in the carbohydrates metabolism. These determinations were made with in vivo oral glucose and sucrose tolerance test and in silico assays. 

#### 2.1.3. Oral Glucose and Sucrose Tolerance Test (OGTT and OSTT) of Ethanolic Extract from *Annona cherimola*, AcRFr, Myricetin, and Rutin

**Figure 2:**
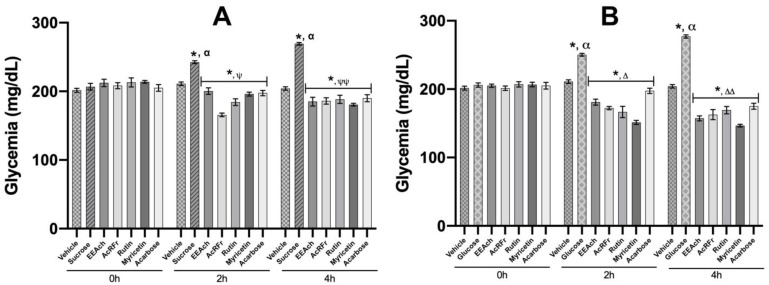
Effect of isolated products from *A. cherimola* on the oral sucrose (OSTT), and glucose tolerance test (OGTT). (**A**) OSTT assay, groups treated with vehicle, sucrose (3 g/kg), EEAch, AcRFr (300 mg/kg), rutin, myricetin, and acarbose (50 mg/kg). (**B**) OGTT assay, groups treated with vehicle, glucose (1.5 g/kg), EEAch, AcRFr (300 mg/kg), rutin, myricetin, and acarbose (50 mg/kg). Data are expressed as means ± SEM, *n* = 6; * *p* < 0.05 vs. initial values; ψ *p* < 0.05 vs. sucrose group 2 h; ψψ *p* < 0.05 vs. sucrose group 4 h; α *p* < 0.05 vs. vehicle 0 h; Δ *p* < 0.05 vs. glucose group 2 h; ΔΔ *p* < 0.05 vs. glucose group 2 h.

In the oral sucrose tolerance test (OSTT) assay, after the administration of EEAch, AcRFr, rutin, and myricetin, a significative reduction of the postprandial peak was observed. All the treatments were significant in comparison with the group treated with sucrose at 2 and 4 h (Figure 2A).

Further, we observed that the group treated with rutin showed lower glycemic levels in comparison with myricetin and acarbose at 2 h of the assay. 

When the oral glucose tolerance test (OGTT) was carried out, the postprandial glucose peak was inhibited in all the treatments administered. The reduction observed was significant in comparison with the group treated with glucose at 2 and 4 h. Moreover, it is important to mention that in this assay, the group treated with myricetin showed lower glycemic levels than the other treatments, inclusive of the control acarbose group (Figure 2B). 

Both results suggest that rutin may have more selectivity to prevent glucose uptake from complex carbohydrate hydrolysis and myricetin from simple carbohydrates. 

### 2.2. In Silico Assays

#### 2.2.1. Molecular Docking Studies of Rutin, Myricetin, and Acarbose on α-Glucosidase Enzyme

To determine the possible interaction of rutin and myricetin, a molecular docking study using as a target the α-glucosidase enzyme was carried out. This enzyme is involved in the hydrolysis of complex carbohydrates such as sucrose. As a control, we used acarbose, an inhibitor of the α-glucosidase enzyme and co-crystalized ligand of the protein 2QMJ. 

The molecular docking analysis showed that rutin has the best affinity to the α-glucosidase enzyme with ΔG values of −6.03 kcal·mol^−1^; it showed ten polar interactions with aminoacidic residues of the enzyme. In the case of myricetin, its affinity values were −3.32 kcal·mol^−1^, with ten polar interactions. Both ligands also have four and three nonpolar interactions, respectively. For acarbose, it showed affinity values of −4.39 kcal·mol^−1^, with sixteen polar and two nonpolar interactions (Table 2).

**Figure 3:**
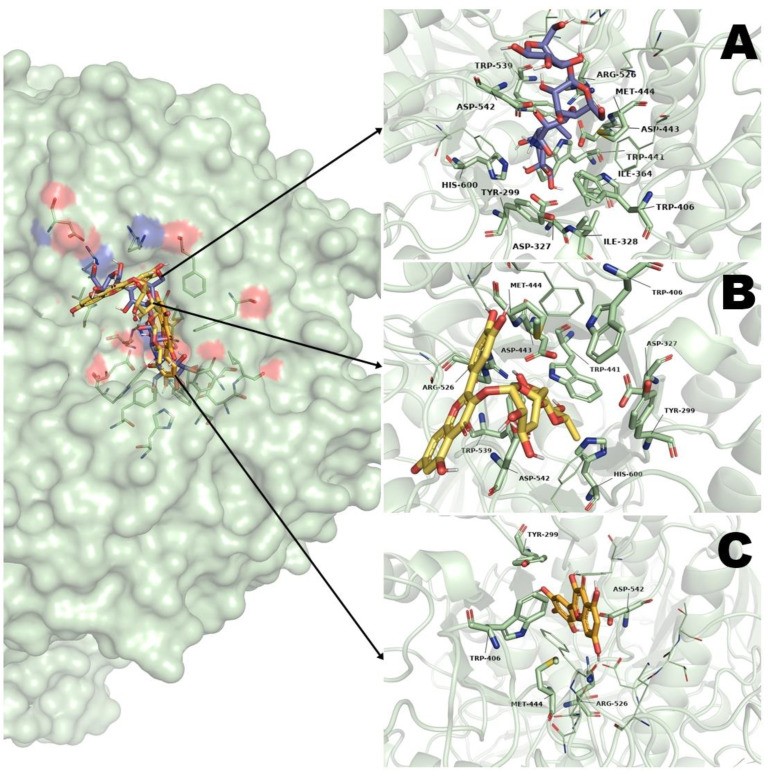
Results of molecular docking on α-glucosidase enzyme. (**A**) Interaction of acarbose and its binding site position; (**B**) Interaction of rutin and its binding site position; (**C**) Interaction of myricetin and its binding site position.

The analysis of the 3D model of binding suggests that the three ligands have the same binding pocket, and their position is similar (Figure 3). It is important to highlight that rutin showed the best affinity to the enzyme than the pharmacological control acarbose.

#### 2.2.2. Molecular Docking Studies of Rutin, Myricetin and Canagliflozin on SGLT1 Cotransporter

The other molecular docking study was carried out using the SGLT1 cotransporter, a protein important involved in the monosaccharide’s absorption on the enterocyte; as a control ligand, canagliflozin was used. 

**Figure 4:**
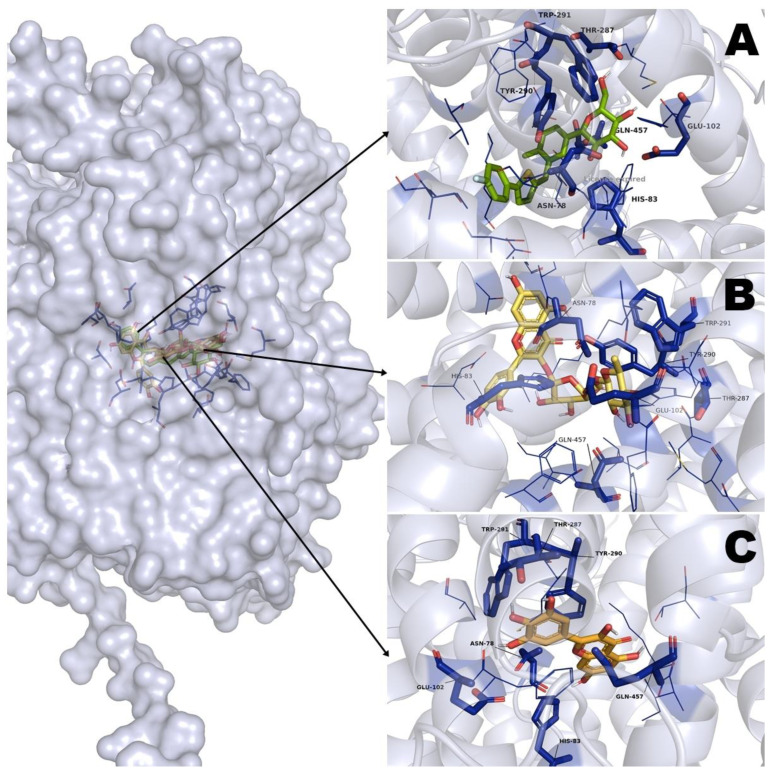
Results of molecular docking on SGLT1 cotransporter. (**A**) Interaction of canagliflozin and its binding site position; (**B**) Interaction of rutin and its binding site position; (**C**) Interaction of myricetin and its binding site position.

The analysis of results from molecular docking showed that rutin has binding free energy (ΔG) values of 22.82 kcal·mol^−1^, with nineteen polar interactions and two nonpolar interactions with the amino acid residues from SGLT1 cotransporter. On the other hand, myricetin showed ΔG values of −7.89 kcal·mol^−1^, with nine polar interactions and four nonpolar interactions. In the case of the pharmacological control canagliflozin, it showed the best ΔG values of −10.08 kcal·mol^−1^, with sixteen polar and six nonpolar interactions (Table 3). The analysis of the 3D of the binding site suggests that the three ligands have the same binding position (Figure 4); however, the ΔG values shown for rutin suggest that this binding position is highly unlikely to take place.

## 3. Discussion

Diabetes mellitus (DM) is a chronic progressive metabolic disorder of carbohydrates, lipids, and proteins. The prevalence of DM continues to increase worldwide, being a constant public health problem [1,2,3]. There are many treatments for DM, and all of them are classified according to their mechanism of action into seven principal groups: insulin sensitizers (thiazolidinediones and biguanides), secretagogues (meglitinides and sulfonylureas), dipeptidyl peptidase-4 (DPP-4) inhibitors, incretin mimetics, glucagon-like peptide-1 (GLP-1) inhibitors, α-glucosidase inhibitors and sodium-glucose cotransporter (SGLT) inhibitors [34,35]. All of them are focused on reducing and controlling hyperglycemia; however, in the long term, some of them can lose their pharmacological activity. Moreover, in all cases, they are accompanied by undesirable side effects for the patient. Insulin sensitizer may cause adverse gastrointestinal effects such as nausea, vomiting, and abdominal discomfort. Secretagogues mostly generate hypoglycemia, DPP-4 inhibitors principally generate gastrointestinal discomfort, incretin mimetics generate hypoglycemia and nausea, GLP-1 inhibitors cause intestinal discomfort, vomiting, and in some cases diarrhea, with respect to a-glucosidase inhibitors, the principal side effect is flatulence generation and abdominal distension, and finally, the SGLT inhibitors can cause urinary tract infections, genital infections and a possible risk of bladder cancer [35,36]. Due to the wide variety of side effects that all treatments of DM can generate, it is necessary to continue searching for new alternatives [4,5,6]. 

The present study aimed to determine the potential antidiabetic activity of an ethanolic extract obtained from the leaves of *Annona cherimola* Miller (*A. cherimola*), one species of the *Annonaceae* family, isolate the minor components from the extract and determine their acute and subchronic activity, as well as to suggest the possible antidiabetic mechanism of action. This study was carried out using activity-guided fractionation as a strategy. 

First, the acute activity of the ethanolic extract from *Annona cherimola* Miller (EEAch) was carried out using female mice with alloxan-induced diabetes type 2 (AIT2D); the results showed that EEAch reduces the hyperglycemic values at 300 mg/kg dose, this was according to other studies that have been made to *A. cherimola*, where several authors have been determined their potential antidiabetic activity in different animal models [28,29,30,31]. To continue the study, the EEAch was fractionated. As a result, the aqueous residual fraction (AcRFr) and dichloromethane fraction (DCMFr) were obtained. 

The aqueous fractions and the dichloromethane fraction were evaluated in the same model, observing that they have an antihyperglycemic effect; however, this effect does not reach the levels of glycemia values of the healthy control group. From both fractions, as observed in Table 1, the fraction that shows a better antihyperglycemic effect is AcRFr, confirming that this fraction contains the compounds responsible for the activity observed with the extract [29]. The next step of the investigation was to isolate the compounds, identify them, and evaluate their activity on the AIT2D model. In other studies, there have been isolated the flavonoid rutin, and this was responsible in part for the antihyperglycemic activity of *A. cherimola*; however, in the EEAch and AcRFr, there are more compounds that need to be studied to search for more alternatives for the treatment of DM. 

The purification of AcRFr led us to the isolation of eight compounds with polyphenolic characteristics. They were identified as seven flavonoids (narcissin, hyperin, nicotiflorin, astragalin, isoquercitrin, rutin, and myricetin) and chlorogenic acid. All the compounds evaluated in the AIT2D model, hyperin, myricetin, nicotiflorin, rutin, isoquercitrin, and narcissin showed a reduction of the hyperglycemic values. Only astragalin and chlorogenic acid did not present antihyperglycemic activity. The activity of the compounds was compared with acarbose, an oral antidiabetic drug (OAD), being more effective in some of the compounds evaluated compared to acarbose (Table 1). The antihyperglycemic activity reported in our study is according to the described by other authors, which indicates that polyphenols are good candidates for the treatment of DM and its complications. The activity of polyphenols as antidiabetic agents varies according to their composition and their substituents [24,37,38,39]. 

Myricetin and rutin were selected as the compounds with the best antihyperglycemic activity due to showing the best reduction of hyperglycemic values; therefore, the study was continued with these compounds. 

Once demonstrated the acute antidiabetic activity of the compounds was, only those with the best antihyperglycemic activity (EEAch, AcRFr, myricetin, and rutin) were evaluated in a subchronic assay using the AIT2D model. After the subchronic evaluation of the products obtained from *A. cherimola*, we observed that EEAch and AcRFr help to reduce hyperglycemic values after 2 weeks of treatment and avoid the exponential increase in hyperglycemic values characteristic of the AIT2D model that normally evolves in DM1 and the hyperglycemic values constantly increase as we can see in Figure 1. This activity is according to that observed in previous studies with *A. cherimola* [30]; this activity is also too observed in other species of the family Annonaceae [40]. 

The groups treated with myricetin and rutin showed a better regulation of hyperglycemic values due to both treatments reaching normoglycemic values from the second week until the end of the treatment. Those activities were similar to those observed in the group treated with acarbose. Our results suggest that myricetin and rutin have a similar activity of acarbose; in this sense, we continue the investigation, and it was proposed to evaluate the possible mechanism of action of both compounds. We propose firstly the evaluation of an α-glucosidase inhibitor due to their having reported activity for rutin [29]; however, myricetin did not have been reported with this activity. Additionally, we propose both compounds be evaluated as an SGLT1 cotransporter. These mechanisms of action were selected due to one alternative to reduce hyperglycemic values that did not generate hypoglycemia is the reduction of postprandial peaks of glucose. In this sense, α-glucosidase enzyme and SGLT1 cotransporters were two proteins involved in the metabolism of complex carbohydrates and absorption of simple carbohydrates, respectively [41,42]. 

In order to determine the potential α-glucosidase and SGLT1 inhibition, two in vivo experiments (OSTT and OGTT) were carried out according to previous investigations [40]. In OSTT, we observed that all treatments reduce the postprandial peak after sucrose load (Figure 2). Further, we observed that the group treated with AcRFr reduced their glycemic values below their initial values and the vehicle control. We hypothesize that in the AcRFr, there are many compounds, and some of them can have activity over other mechanisms of action that, added to the α-glucosidase inhibition, have a synergic activity and generate the hypoglycemia observed. In the case of rutin, we observed that the activity of this compound is better than the pharmacological control acarbose; this is according to the described in the literature, which indicates that rutin is a flavonoid that avoids the postprandial peak after a sucrose load in rat and mice models [22,25]. 

When the OGTT test was carried out, we observed a similar activity to the OSTT, i.e., all treatments significantly reduced the glucose postprandial peak. Moreover, we observed that in this case, all the compounds obtained from *A. cherimola* showed to have more activity than the pharmacological control acarbose. In the case of rutin and myricetin, we observed that it seems that myricetin has more activity in OGTT than rutin due to it perhaps having more selective activity directed to SGLT1 cotransporter that is involved in the glucose absorption [43]. Once demonstrated the in vivo activity over the carbohydrates metabolism, we decided to carry out in silico studies to determine computationally the results obtained in OSTT and OGTT and to support what we observed with the in vivo assays. 

Finally, to corroborate the results obtained in OGTT and OSTT, molecular docking studies were carried out to determine the possible binding sites of rutin and myricetin in α-glucosidase enzyme and SGLT1 cotransporter. 

Regarding α-glucosidase enzyme, rutin showed a ∆G value of −6.03 kcal·mol^−1^, myricetin a ∆G value of −3.32 kcal·mol^−1^ and acarbose a ∆G value of −4.39 kcal·mol^−1^ (Table 2). Our result agrees with other in silico studies that indicate a better binding ∆G value for rutin in comparison with acarbose, an α-glucosidase inhibitor [25]. Moreover, rutin shares with acarbose ten (Tyr 299, Asp 327, Trp 406, Trp 441, Asp 443, Met 444, Arg526, Trp 539, Asp 542, His 600) of the eleven (Tyr 299, Asp 327, Ile 328, Trp 406, Trp 441, Asp 443, Met 444, Arg526, Trp 539, Asp 542, His 600) aminoacidic residues important in the binding site of α-glucosidase enzyme (Table 2) [44,45]. In the case of myricetin, this molecule only shares five (Tyr299, Trp406, Met444, Arg526, Asp542) aminoacidic residues with acarbose. The 3D binding visualization (Figure 3) showed that the three ligands are posed in the same binding pocket over the surface of α-glucosidase enzyme; however the only difference between rutin and myricetin binding is that rutin showed a major number of polar interactions, these interactions were carried out mainly by the di-glycoside in rutin.

Regarding molecular docking using the SGLT1 cotransporter, we observed an important result. Rutin showed a ΔG value of 22.82 kcal·mol^−1^, myricetin ΔG values of −7.89 kcal·mol^−1,^ and canagliflozin ΔG values of −10.08 kcal·mol^−1^. Myricetin shares seven aminoacidic residues (Asn78, His83, Glu102, Thr287, Tyr290, Trp291, Gln457) with canagliflozin, an inhibitor of SGLT1, is important to mention that these aminoacidic residues are important for the inactivation of SGLT1 due to there is where glucose binds to be transported over the SGLT1 [37,46]. In the case of rutin, it shares only three aminoacidic residues (Phe 101, Tyr 290, Gln457) with acarbose. However, we observed that the ΔG value of this ligand was 22.82 kcal·mol^−1^; this value indicates that it is unlikely that rutin takes place on this binding position, due to positive ΔG values means that there is a need for a lot of energy to generate that binding position. Our analysis suggests that there is a steric hindrance with rutin that does not let the molecule interact with the binding pocket in SGLT1. In this case, we suggest that it is probably due to the rutin molecule having di-gycoside; this perhaps generates this steric hindrance, and that is why this union position is unlikely. 

We suggest this analysis since in carbohydrates metabolism, once hydrolyzed, the disaccharides to monosaccharides can be transported from the small intestine to the bloodstream via a complex mechanism mediated by SGLT1/SLC5A1 cotransporters and glucose transport facilitating systems (GLUTs) [40], in this sense, di-glycosides cannot be transported by SGLT either GLUT-2. 

The complete analysis of our investigation provides new information on the minor compounds present in the aqueous residual fraction from *A. cherimola*, their antihyperglycemic activity, the possible mechanism of action of two of the most active compounds isolated from the aqueous residual fraction (rutin and myricetin), that according to the results showed, it can be mediated by the inhibition of α-glucosidase enzyme and SGLT-1 cotransporter. Moreover, this research could lay the foundations for the development of new therapies for the treatment of DM based on compounds of a flavonoid nature.

## 4. Materials and Methods

### 4.1. Chemicals, Reagents, and Drugs

Alloxan monohydrate (PN: A7413-25g), Acarbose (PN: PHR1253-500MG), glibenclamide (PN: PHR1287-1G), metformin (PN: PHR1084-500MG), glucose (anhydrous, PN: D9434-1Kg), sucrose (≥99.5% GC, PN: S9378-1Kg) were purchased from Sigma-Aldrich^®^ (Sigma^®^, Saint Louis, MO, USA), Ethanol anhydrous (Catalogue code: 15568604), ethyl acetate (CC:10382681), methanol (CC:10284580), and dichloromethane (CC:15594055) solvents were purchased from J.T. BakerTM (Thermo Fisher Scientific, Waltham, MA, USA), TLC glass plates L × W 20 cm × 20 cm, sílica gel 60 F254, 2 mm (CC: Z292974) were purchased from Merck^®^ (Merck^®^, Darmstadt, Germany). 

### 4.2. Plant Material

*Annona cherimola* Miller leaves were collected by Dr. Fernando Calzada in December 2019 at San Jose, Tláhuac, Mexico (19°16′32.6″ N 99°00′07.1″ W). A sample was authenticated by the botanist MSc Abigail Aguilar Contreras of the Medicinal Plant Herbarium (IMSSM) in the Mexican Institute of Social Security (IMSS) and stored for further reference under the number: 15,795. Leaves were washed twice with distilled water and left to dry in the dark for seven days long at room temperature, then were grounded with an electric grinder (model M-22-RW, Fundacion Torrey, Apodaca, Nuevo León, México). 

### 4.3. Extraction, Isolation, and Identification of Minor Components from Annona cherimola

The grounded leaves from *A. cherimola* (2.9 kg) were macerated at room temperature in ethanol (10 L × 2 times). The resulting extract was filtrated through grade 1 filter paper and concentrated at 40 °C under reduced pressure with a rotary evaporator (Büchi Labortechnik AG, Flawil, Switzerland). At the end of the extraction procedure, 131 g of a dark green sticky precipitate was obtained and stored at 4 °C to be used after. The yield of the extraction per gram of vegetable powder was evaluated by the difference in the weight of the container before and after pouring the extract, obtaining 4.51% after extraction.

The extract was fractioned, and a portion of EEAch (40 g) was suspended in 10% EtOH-water (100 mL) and successively portioned with dichloromethane (100 mL × 3) to obtain 25 g DCMFr. The aqueous residual layer was collected to obtain 15.1 g of aqueous residual fraction (AcRFr). The activity was associated with AcRFr, then a portion (300 mg) was purified by preparative thin layer chromatography (silica gel 60F-254 Merck), using EtOAc:MeOH: water (10:1.6:1.3) mixture to obtain astragalin (12.73 mg), chlorogenic acid (3.41 mg), hyperin (36.61 mg), isoquercitrin (20.1 mg), myricetin (14.38 mg), narcissin (37.19 mg), nicotiflorin (34.14 mg), and rutin (73.54 mg), the compounds were identified by NMR ^1^H and ^13^C (see Supplementary Materials); further, the compounds were compared with standards. The process was repeated as needed.

### 4.4. In Vivo Assays

#### 4.4.1. Animals

In order to evaluate the biological activity, female BALB/c mice of 20 ± 5 g body weight (bw) (aged 8–10 weeks) were obtained from the Animal Facility from the Animal House of the National Medical Center “Siglo XXI” from Instituto Mexicano del Seguro Social (IMSS), housed under standard laboratory conditions at a temperature of 22 ± 2 °C, 50% of humidity with a 12 h light/dark cycle and fed with a standard diet 5001 (Lab Diet ^®^, Saint Louis, MO, USA) also purified water ad libitum. This study was approved by the Specialty Hospital Ethical Committee of the National Medical Center “Siglo XXI” from IMSS, with the registered numbers: R-2020-3601-038 and R-2019-3601-004. All the experimental animal models used in this study were performed following the Mexican Official Regulations on animal care and experimental management [47].

#### 4.4.2. Induction of Experimental Type 2 Diabetes

The experimental diabetes mellitus was induced through the administration of Alloxan at 74 mg/kg intraperitoneally (IP) in mice previously fasted for 24 h. Alloxan was dissolved in injectable water at room temperature, and 0.5 mL of this solution was administrated twice in each animal. After the induction, all animals were allowed to drink a 10% sucrose aqueous solution to stabilize the induced hyperglycemia. Then, 24 h after, the development of SIT2D was determined by measuring postprandial blood glucose levels using a conventional glucometer (ACC CHECK^®^ Performa Blood Glucose Systems, Roche^®^, DC, Basel, Switzerland).

Additionally, to confirm the AIT2D model, the β-cell function was evaluated with the administration of 5 mg/kg glibenclamide orally and measuring the decrease in glucose values 2 and 4 h after administration; according to the results, there can be confirmed the existence of functional β-cell [40]; therefore, the generated model was classified as an experimental type 2 diabetes mellitus model.

#### 4.4.3. Grouping

For the evaluations of the antidiabetic effect of the ethanolic extract, BALB/c mice were randomly divided into groups of 6 each as follows: ethanolic extract of *A. cherimola* (EEAch) at the dose of 300 mg/kg, aqueous fraction (AcRFr) at 300 mg/kg, and dichloromethane fraction (DCMFr). In the case of the compounds isolated from the EEAch, the dose used was 50 mg/kg in all cases. Moreover, the oral antidiabetic drug (OAD) metformin acarbose (Aca) at 50 mg/kg bw was used as the positive control. Further, normoglycemic and AIT2D control groups were carried out. In all cases, the treatments were dissolved in 2% Tween 80 in water as vehicle and were given orally through a gavage; the volume was calculated according to international guidelines [48] as 2 mL/100 g body weight.

#### 4.4.4. Acute Evaluation of the Antidiabetic Effect of Ethanolic Extract from *Annona cherimola*, Fractions, and Isolated Compounds

Animals with blood glucose levels between 190–220 mg/dL were used for this study. The treatments described above were administered orally; once the treatments were administered, the blood samples were collected from the tail vein at the beginning (0 h), 2 and 4 h after administration using a conventional glucometer described previously. 

#### 4.4.5. Subchronic Evaluation of the Antidiabetic Effect of Ethanolic Extract from *Annona cherimola*, Fractions, and Isolated Compounds

Experimental-induced diabetes type 2 animals in the same conditions previously described were used for the subchronic evaluation. In this case, the animals were administered with the treatments as described above once daily for 4 weeks. Blood glucose levels were measured weekly, as previously described.

#### 4.4.6. Oral Glucose and Sucrose Tolerance Test (OGTT and OSTT) of Ethanolic Extract from *Annona cherimola*, Fractions, and Isolated Compounds

The oral sucrose tolerance test (OSTT) was conducted according to published protocols [36]. Diabetic fasted female mice were randomly divided into eight groups of 6 animals each as follows: control group treated only with water as vehicle, EEAc (300 mg/kg), AcRFr (300 mg/kg), DCMFr (300 mg/kg), myricetin (50 mg/kg), rutin (50 mg/kg), and acarbose (50 mg/kg) as pharmacological control. In order to perform the test, the time 0 was set before treatments; then, treatments were given orally, and 30 min after the administration, a sucrose load (3 g/kg) was administered to the mice. Measures of the blood glucose level from the tail vein were performed at 2 and 4 h after administration of the carbohydrate by applying the glucose oxidase method using a standard glucometer (Accu-Chek^®^ Performa Glucometer, Boehringer Mannheim, Germany) [49]. The oral glucose tolerance test (OGTT) was performed under the same conditions as the OSTT assay, but in this case, a glucose load (1.5 g/kg) was given to the groups, and the blood glucose measurements were recorded following the same method for the OGTT. 

### 4.5. In Silico Studies

The chemical structure of ligands rutin (CID: 5280805), myricetin (CID: 5281672), canagliflozin (CID: 24812758), and acarbose (CID: 41774) were retrieved from the chemical library PubChem (https://pubchem.ncbi.nlm.nih.gov/) (accessed on 12 December 2022), these were optimized and submitted to energetic and geometrical minimization using Avogadro software [50]. The α-glucosidase enzyme (crystal structure of the N-terminal of Human Maltase–Glucoamylase, RCSB, PDB ID: 2QMJ) and SGLT-1 cotransporter (crystal structure of human sodium/glucose cotransporter, Uniprot ID: P13866) were used as a target of the study. These were retrieved from the Protein Data Bank (http://www.rcsb.org/) (accessed on 12 December 2022) and the Uniprot database (https://www.uniprot.org/) (accessed on 12 December 2022). Total molecules of water and ions not needed for catalytic activity were stripped to preserve the entire protein. All polar hydrogen atoms were added and ionized in a basic environment (pH = 7.4), and Gasteiger charges were assigned; the computed output topologies from the previous steps were used as input files to docking simulations.

The molecular docking experiments were carried out using Autodock 4.2 software [51]; the search parameters were as follows: a grid-base procedure was employed to generate the affinity maps delimiting a grid box of 126 × 126 × 126 Å^3^ in each space coordinate, with a grid points spacing of 0.375 Å, the Lamarckian genetic algorithm was employed as scoring function with a randomized initial population of 100 individuals and a maximum number of energy evaluations of 1 × 10^7^ cycles, the analysis of the interactions in the enzyme/inhibitor complex was visualized with PyMOL software (The PyMOL Molecular Graphics System, Ver 2.0, Schrödinger, LLC, DeLano Scientific, San Carlos, CA, USA).

The validation of the molecular docking was carried out by re-docking the co-crystallized ligand in the proteins (α-glucosidase and SGLT-1). The lowest energy pose of the co-crystallized ligand was superimposed, and it was observed whether it maintained the same bind position. The RMSD was calculated, and a reliable range within 2 Å is reported.

### 4.6. Statistical Analysis

All the results are expressed as mean values standard error of the mean (SEM). All statistical analyses were performed using GraphPad Prism version 8.2.1 (GraphPad Software Inc., San Diego, CA, USA). The statistical evaluation was conducted through an analysis of variance (ANOVA) followed by a Tukey test for multiple comparisons considering the *p*-value ≤ 0.05 was a statistically significant difference.

## 5. Conclusions

The acute and subchronic in vivo evaluations showed that in the current AIT2D model, EEAch, AcRFr, DCMFr, rutin, and myricetin reduced the hyperglycemic values. The oral glucose and sucrose tolerance test and the molecular docking suggest that the antidiabetic activity of the products obtained from *A. cherimola* is mediated in part by α-glucosidase enzyme inhibition and SGLT1 cotransporter inhibition. 

This research supports the phytochemical and pharmacological bases of *A. cherimola* and its use as a source for the development of new potential antidiabetic agents for T2D control based on flavonoid molecules. 

## Figures and Tables

**Table 1:** Blood glucose levels of normoglycemic mice (NM) and alloxan-induced type 2 diabetic mice (AIT2D) at 0, 2, and 4 h, on the acute antihyperglycemic test.

Treatment	Glycemia (mg/dL)		
	0 h	2 h	4 h
NM Control	154.3 ± 0.4	151.3 ± 1.2	146.6 ± 2.1
AIT2D Control	214.0 ± 5.7	218.5 ± 3.7	221.5 ± 3.9
AIT2D + EEAch	205.0 ± 4.0	143.7 ± 2.4 *^,Δ^	134.0 ± 5.1 *^,ΔΔ^
AIT2D + AcRFr	204.0 ± 2.8	163.7 ± 3.6 *^,Δ^	174.7 ± 0.8 *^,ΔΔ^
AIT2D + DCMFr	207.0 ± 5.1	181.0 ± 0.8 *^,Δ^	189.0 ± 0.4 *^,ΔΔ^
AIT2D + Astragalin	203.3 ± 4.8	190.7 ± 1.3 ^Δ^	200.0 ± 2.6 ^ΔΔ^
AIT2D + Chlorogenic acid	171.0 ± 12.5	193.0 ± 6.4 ^Δ^	211.0 ± 3.4 ^ΔΔ^
AIT2D + Hyperin	203.0 ± 1.4	152.7 ± 5.5 *^,Δ^	184.0 ± 5.3 *^,ΔΔ^
AIT2D + Isoquercitrin	206.3 ± 5.1	169.0 ± 3.2 *^,Δ^	197.7 ± 3.6 ^ΔΔ^
AIT2D + Myricetin	197.3 ± 2.5	161.0 ± 6.2 *^,Δ^	155.0 ± 3.1 *^,ΔΔ^
AIT2D + Narcissin	204.7 ± 4.0	186.0 ± 4.1 ^Δ^	167.3 ± 6.3 *^,ΔΔ^
AIT2D + Nicotiflorin	206.7 ± 0.3	176.7 ± 2.1 *^,Δ^	179.7 ± 2.4 *^,ΔΔ^
AIT2D + Rutin	214.3 ± 4.0	170.6 ± 8.3 *^,Δ^	162.3 ± 3.6 *^,ΔΔ^
AIT2D + Acarbose	205.0 ± 1.6	190.0 ± 2.0 *^,Δ^	181.3 ± 3.0 *^,ΔΔ^

EEAch, AcRFr, and DCMFr were administered at 300 mg/kg; astragalin, chlorogenic acid, hyperin, isoquercitrin, myricetin, narcissin, nicotiflorin rutin, and acarbose were administered at 50 mg/kg. Data are expressed as means ± SEM, *n* = 6; * *p* < 0.05 vs. initial values; Δ *p* < 0.05 vs. SIT2D control for 2 h; ΔΔ *p* < 0.05 vs. SIT2D control for 4 h SEM: standard error of the mean; NM: normoglycemic mice; AIT2D: alloxan-induced type 2 diabetes mice; EEAch: ethanolic extract of *Annona cherimola* Miller; AcRFr: aqueous residual fraction; DCMFr: dichloromethane fraction.

**Table 2:** Binding energy and interactions of ligands rutin, myricetin, and acarbose on α-glucosidase enzyme.

Ligand	α-Glucosidase			
	ΔG (kcal·mol^−1^)	H-BR	NPI	RMSD
Rutin	−6.03	Asp203, Asp327, Trp441, Met444, Ser448, Phe450, Arg526, Trp539 Asp542, His600	Tyr299, Trp406, Asp443, Phe575,	-
Myricetin	−3.32	Asp203, Thr204, Thr205, Trp406, Met444, Ser448, Asn449, Phe450, Arg526, Asp542	Tyr299, Lys480, Phe575	-
Acarbose	−4.39	Arg202, Thr204, Tyr299, Asp327, Ile328, Ile364, Trp441, Asp443, Phe450, Asp474, Lys480, Arg526, Trp539, Asp542, Phe575, His600	Trp406, Met444	1.93

**Table 3:** Binding energy and interactions of ligands rutin, myricetin and acarbose on SGLT1 cotransporter.

Ligand	Sodium-Glucose Cotransporter (SGLT1)			
	ΔG (kcal·mol^−1^)	H-BR	NPI	RMSD
Rutin	22.82	Ser77, Asn78, His83, Phe101, Glu102, Ala105, Lys157, Gly282, Met283, Leu286, Thr287, Trp289, Tyr290, Lys321, Ser393, Ile397, Gln457, Thr460, Leu527	Asp161, Ile456	-
Myricetin	−7.89	Asn78, His83, Phe101, Glu102, Met283, Thr287, Tyr290, Gln457, Thr 460	Ala105, Lys157, Trp291, Ile456	-
Canagliflozin	−10.08	Thr56, Ser77, His83, Phe101, Glu102, Asp161, Met283, Thr287, Trp289, Trp291, Ser393, Ser396, Phe453, Gln457, Thr460	Asn78, Lys157, Tyr290, Val296, Ile397, Ile456	1.37

## Data Availability

Not applicable.

## Supplementary Materials

The following supporting information can be downloaded at: https://www.mdpi.com/article/10.3390/ph16050724/s1.
