# Supplementary material for: Annona cherimola Miller and Its Flavonoids, an Important Source of Products for the Treatment of Diabetes Mellitus: In Vivo and In Silico Evaluations
Source: Pharmaceuticals (Basel). 2023 May 10;16(5):724. doi: 10.3390/ph16050724 (PMC10222912; doi:10.3390/ph16050724)
Supplement: Supplementary file 1 [file pharmaceuticals-16-00724-s001.zip › pharmaceuticals-2344064-supplementary.pdf]

Supplementary material (pharmaceutical-2344064)

Title:

Annona cherimola Miller and Its Flavonoids, an Important Source of Products for the Treatment of Diabetes Mellitus: In Vivo and In Silico Evaluations

Fernando Calzada [1,*], Miguel Valdes [2,*], Jesús Martínez-Solís [2], Claudia Velázquez[3] and Eliazbeth Barbosa[2]

Institutions:

[1] Unidad de Investigación Médica en Farmacología, UMAE Hospital de Especialidades 2º Piso CORSE, Centro Médico Nacional Siglo XXI, Instituto Mexicano del Seguro Social, Av. Cuauhtémoc, 330, Col. Doctores, Mexico City, CP 06720, Mexico.

[2] Instituto Politécnico Nacional, Sección de Estudios de Posgrado e Investigación, Escuela Superior de Medicina, Plan de San Luis y Salvador Díaz Mirón S/N, Col. Casco de Santo Tomás, Miguel Hidalgo, Mexico City, CP 11340, Mexico.

[3] Área académica de Farmacia, Instituto de Ciencias de la Salud, Universidad Autonoma del Estado de Hidalgo, Circuito exHacienda La Concepcion s/n, Carretera Pachuca- Atocpan, CP 42160, San Agustin Tlaxiaca Pachuca, Hidalgo, Mexico.

*Correspondence: fercalber10@gmail.com (F.C.); valdesguevaramniguel@gmail.com (M.V.)

**Table of contents**	**Page**
^{1}H-NMR of Astragalin	2
^{1}H-NMR of Chlorogenic acid	3
^{1}H-NMR of Hyperin	4
^{1}H-NMR of Isoquercitrin	5
^{1}H-NMR of Myricetin	6
^{1}H-NMR of Narcissin	7
^{1}H-NMR of Nicotiflorin	8
^{1}H-NMR of Rutin	9
^{13}C-NMR of Astragalin	10
^{13}C-NMR of Chlorogenic acid	11
^{13}C-NMR of Hyperin	12
^{13}C-NMR of Isoquercitrin	13
^{13}C-NMR of Myricetin	14
^{13}C-NMR of Narcissin	15
^{13}C-NMR of Nicotiflorin	16
^{13}C-NMR of Rutin	17

^{1}H-NMR of Astragalin

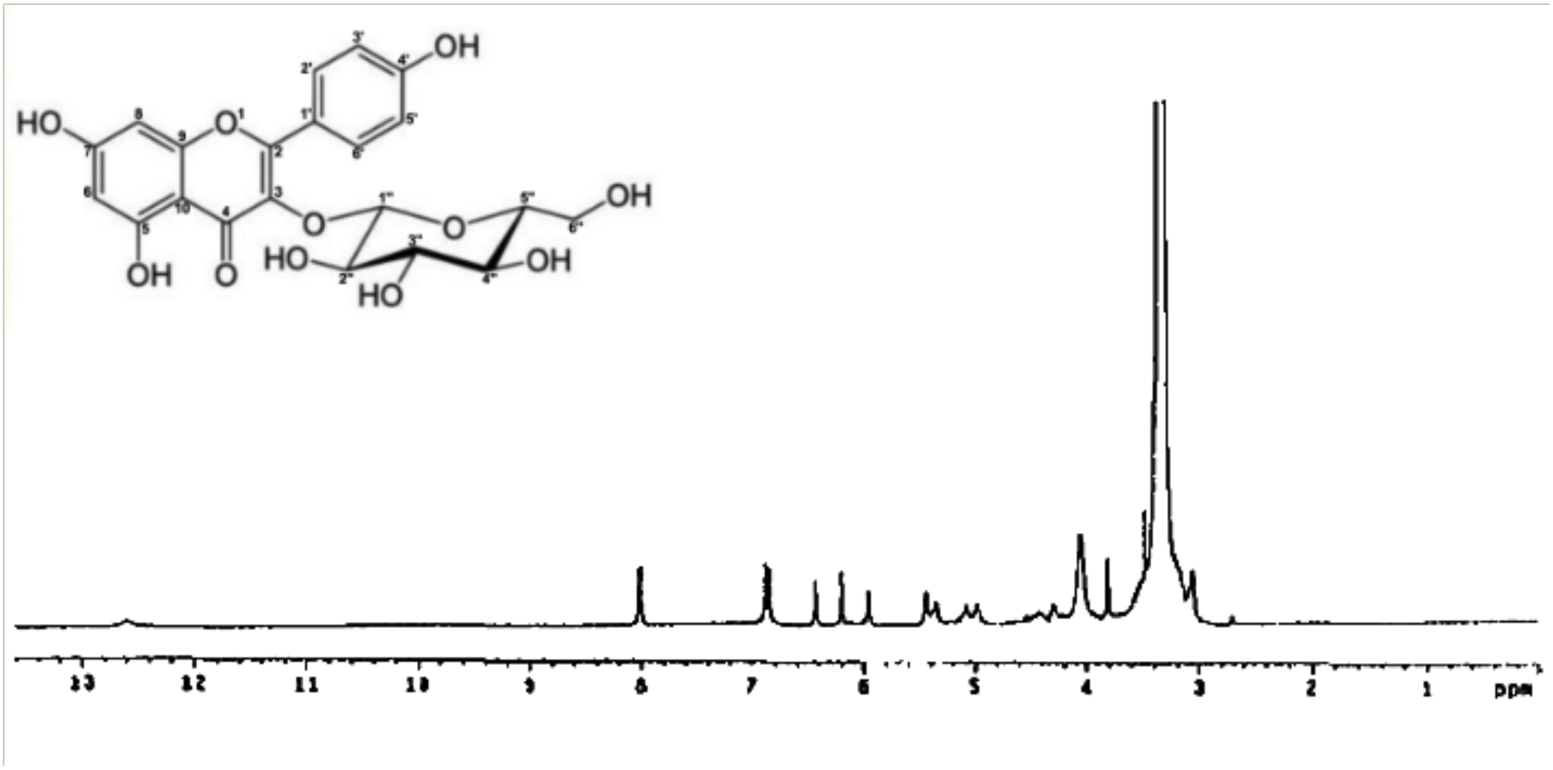

Position	Astragalin
	δH (int, mult., J (Hz))
1	-
2	-
3	-
4	-
5	-
6	6.22 (1H, s)
7	-
8	6.41 (1H, s)
9	-
10	-
1’	-
2’,6’	8.12 (2H, d, 8.5)
3’,5’	6.85 (2H, d, 8.5)
4’	-
1’’	5.26 (1H, d, 7.2)
2’’	3.46 (1H, dd, 10.4)
3’’	3.37 (1H, m)
4’’	3.24 (1H, m)
5’’	3.56 (1H, m)
6’’	3.70 (2H, d, 11.5)

^{1}H-NMR of Chlorogenic acid

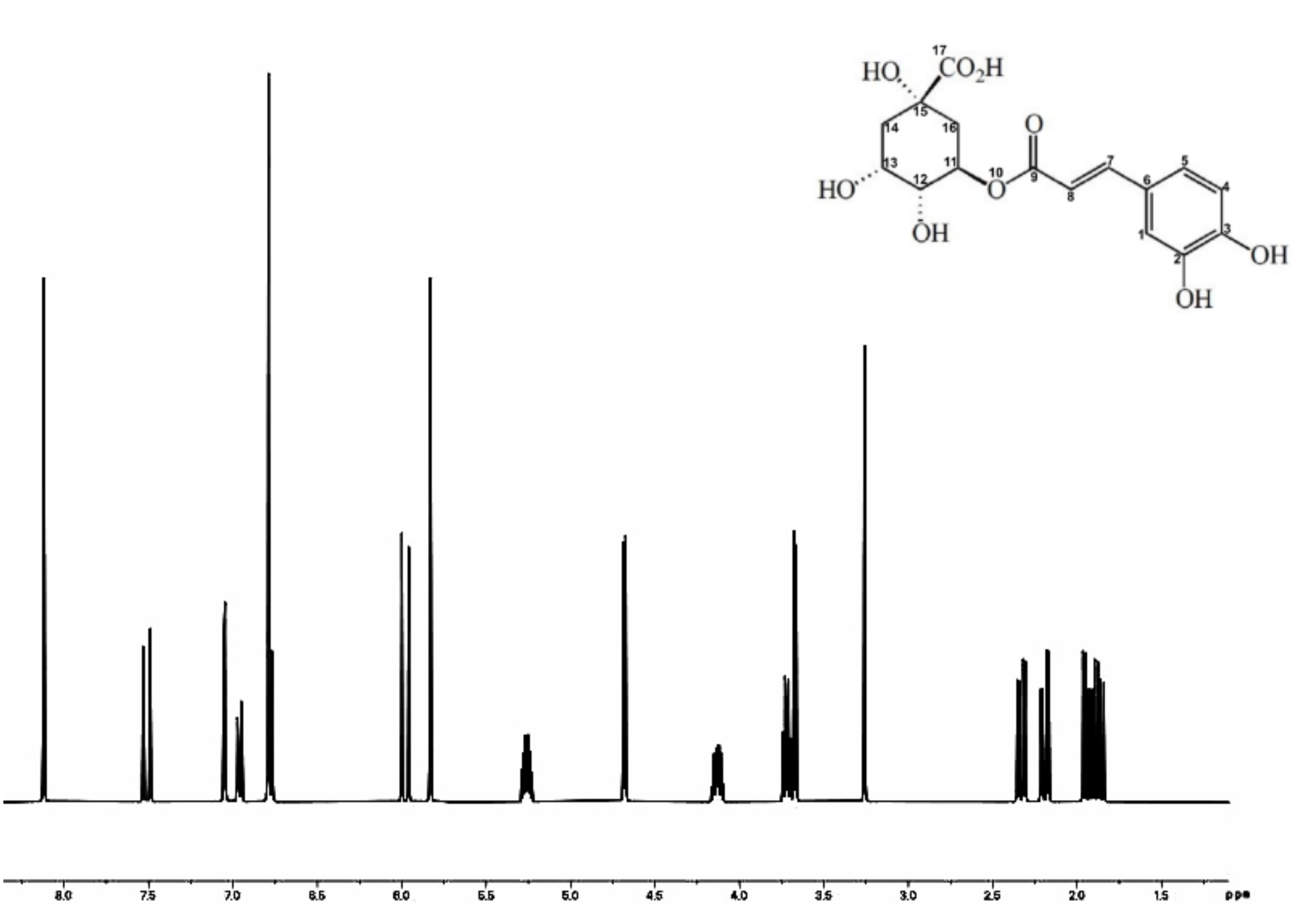

Position	Chlorogenic Acid<br>δH (int, mult., J (Hz))
1	7.05 (1H, d, 1.82)
2	-
3	-
4	6.78 (1H, s)
5	6.96 (1H, ddd, 2.19)
6	-
7	7.51 (1H, dd, 2.01)
8	5.98 (1H, d, 1.90)
9	-
10	-
11	5.26 (1H, ddt, 2.19)
12	3.72 (1H, dt, 2.39)
13	4.13 (1H, dqd, 2.32)
14	2.5 (2H, dd, 2.33)
15	-
16	1.99 (4H, m, 1.91)
17	-

^{1}H-NMR of Hyperin

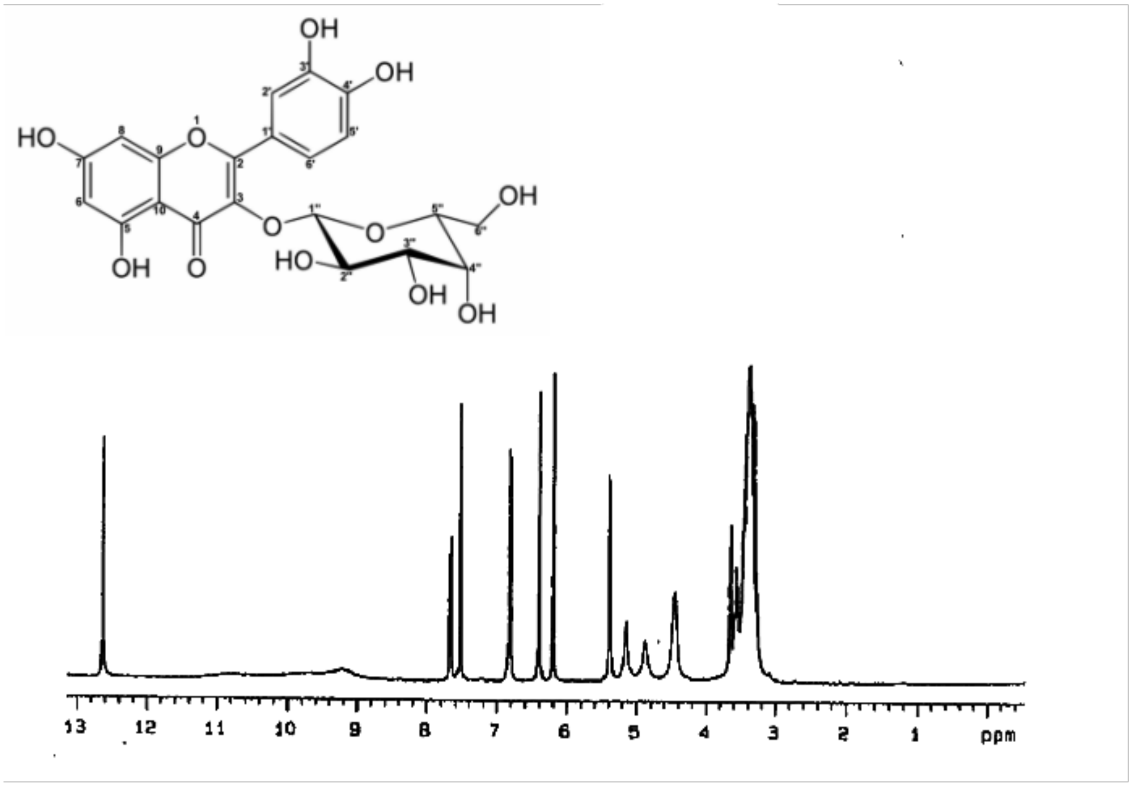

Position	Hyperin
	δH (int, mult., J (Hz))
1	-
2	-
3	-
4	-
5	-
6	6.21 (1H, d, 1.00)
7	-
8	6.37 (1H, d, 1.00)
9	-
10	-
1’	-
2’	7.62 (1H, m, 1.00)
3’	-
4’	-
5’	6.78 (1H, s)
6’	7.50 (1H, dd, 2.01)
1’’	5.46 (1H, m, 2.01)
2’’	3.54 (1H, m, 2.03)
3’’	3.40 (1H, m, 2.05)
4’’	3.52 (1H, m, 1.02)
5’’	3.34 (1H, m, 2.03)
6’’	3.60 (2H, m, 1.01)

^{1}H-NMR of Isoquercitrin

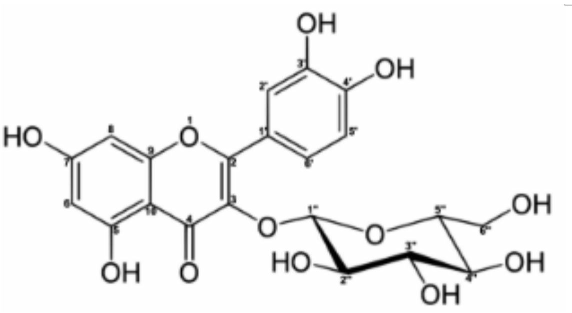

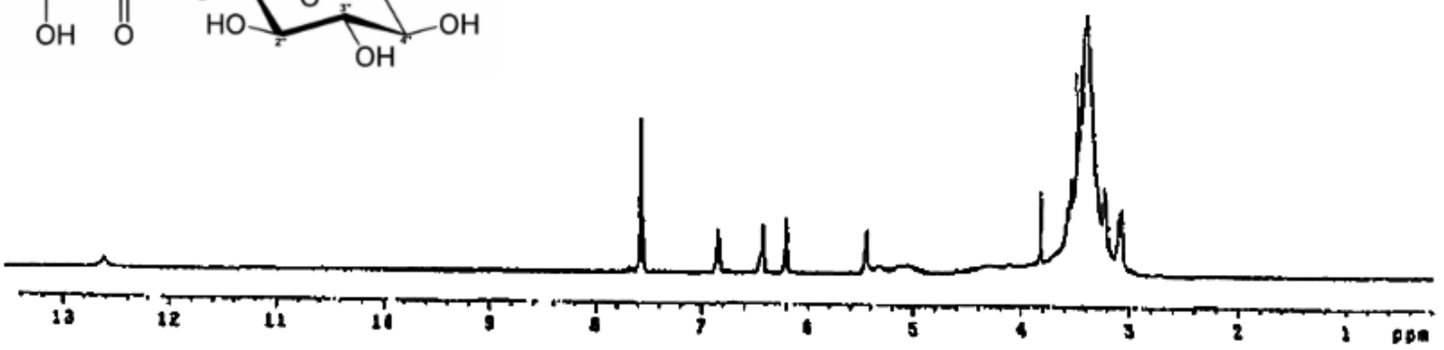

Position	Isoquercitrin
	δH (int, mult., J (Hz))
1	-
2	-
3	-
4	-
5	-
6	6.21 (1H, d, 1.03)
7	-
8	6.37 (1H, d, 1.00)
9	-
10	-
1’	-
2’	7.62 (1H, m, 1.02)
3’	-
4’	-
5’	6.78 (1H, s)
6’	7.50 (1H, dd, 2.01)
1’’	5.46 (1H, ddd, 1.01)
2’’	3.54 (1H, m, 2.02)
3’’	3.21 (1H, dddd, 1.02)
4’’	3.08 (2H, dd, 2.03)
5’’	3.08 (2H, dd, 2.03)
6’’	3.81 (2H, dtd, 2.02)

^{1}H-NMR of Myricetin

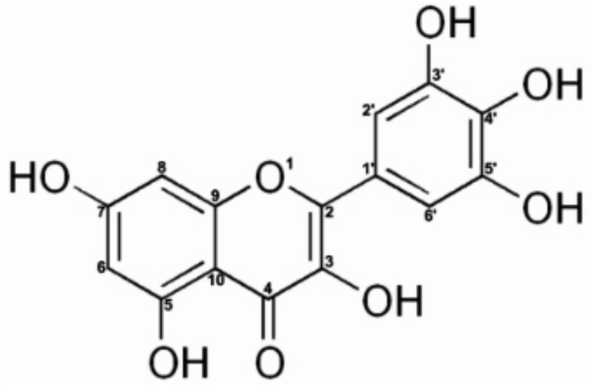

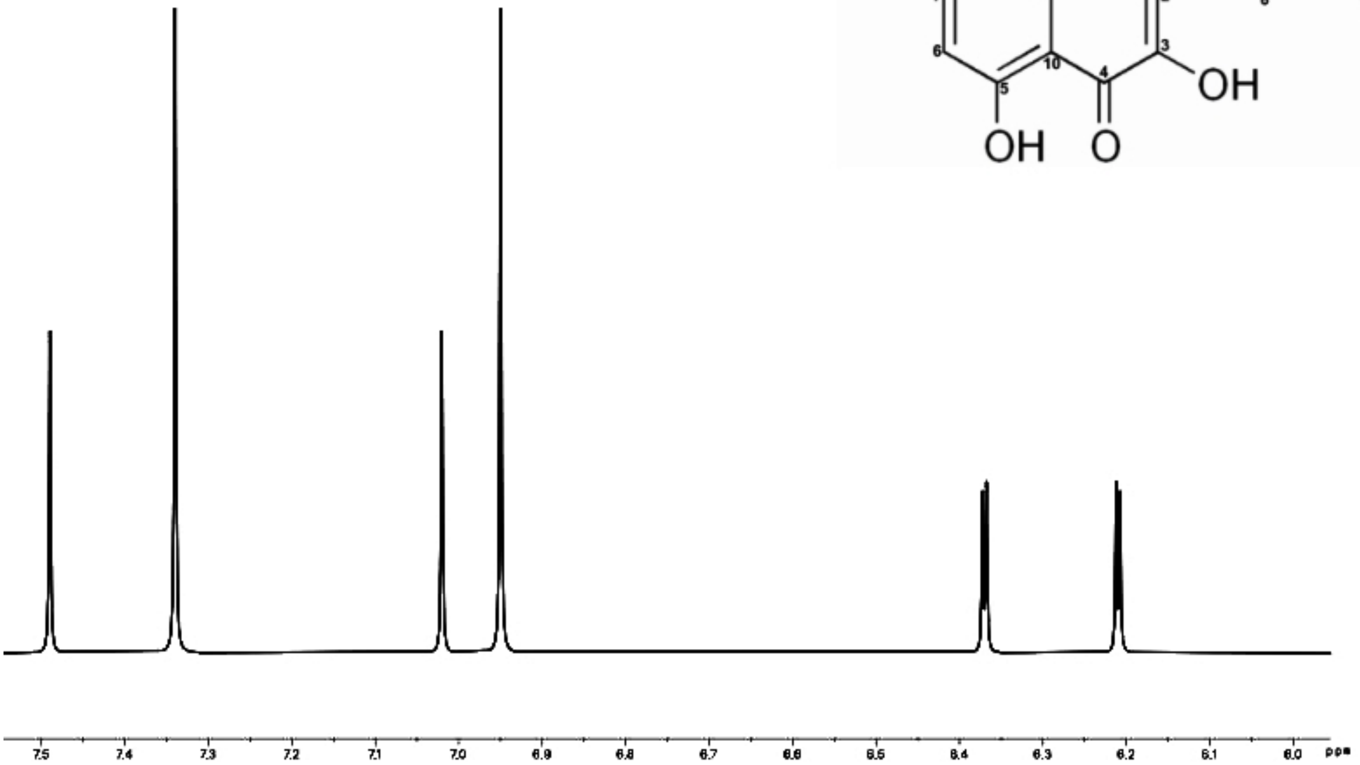

Position	Myricetin
	δH (int, mult., J (Hz))
1	-
2	-
3	-
4	-
5	-
6	6.21 (1H, d, 1.11)
7	-
8	6.37 (1H, d, 1.16)
9	-
10	-
1’	-
2’	6.95 (2H, s)
3’	-
4’	-
5’	-
6’	6.95 (2H, s)

^{1}H-NMR of Narcissin

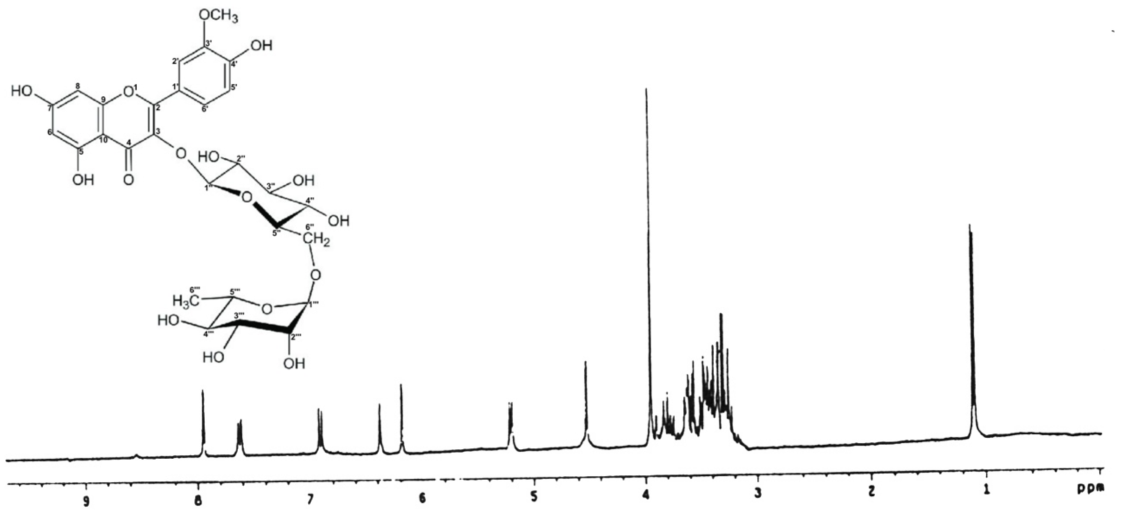

Position	Narcissin
	δH (int, mult., J (Hz))
1	-
2	-
3	-
4	-
5	-
6	6.22 (1H, d, 0.96)
7	-
8	6.87 (1H, d, 0.86)
9	-
10	-
1’	-
2’	8.16 (1H, d, 0.84)
3’	-
OCH_3	3.96 (3H, s)
4’	-
5’	6.15 (1H, s)
6’	7.84 (1H, dd, 0.97)
1’’	5.34 (1H, dt, 0.99)
2’’	3.55 (2H, m, 2.04)
3’’	3.32 (2H m, 1.69)
4’’	3.92 (1H, m, 2.22)
5’’	3.13 (1H, dtq, 0.85)
6’’	3.69 (2H, dd, 1.96)
1’’’	4.54 (1H, m, 1,87)
2’’’	3.76 (2H, m, 1.96)
3’’’	3.76 (2H, m, 1.96)
4’’’	3.32 (2H m, 1.69)
5’’’	3.55 (2H, m, 2.04)
6’’’	1.26 (3H, dd, 2.89)

^{1}H-NMR of Nicotiflorin

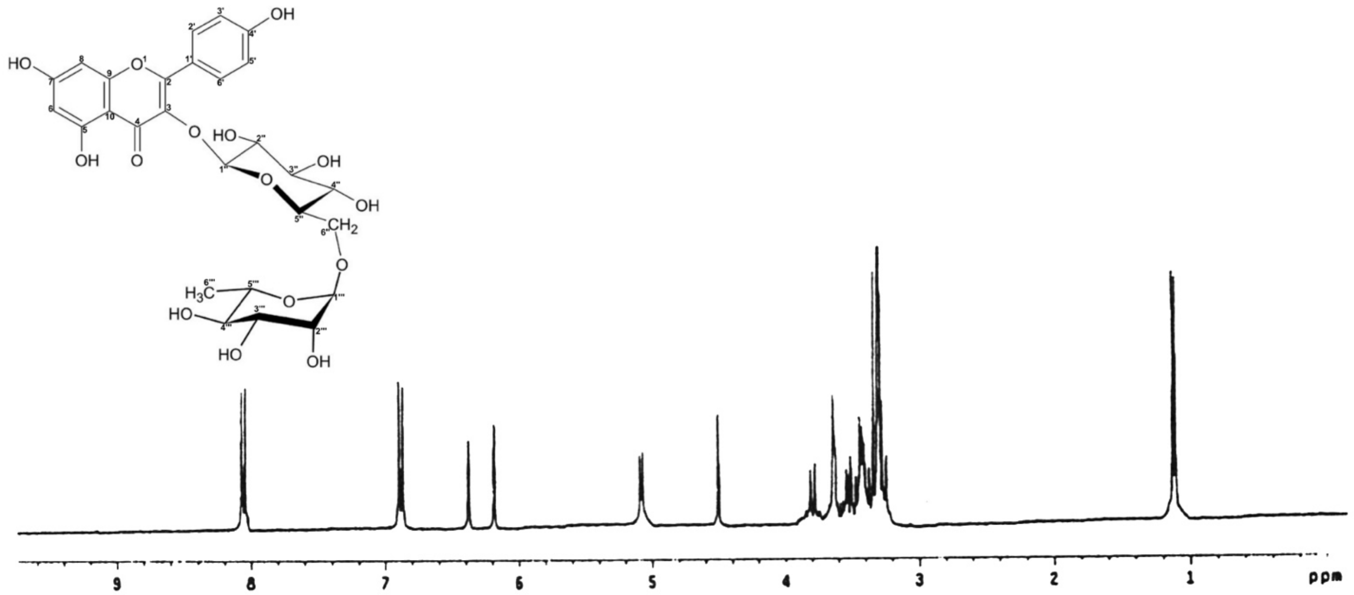

Position	Nicotiflorin δH (int, mult., J (Hz))
1	-
2	-
3	-
4	-
5	-
6	6.21 (1H, d, 0.64)
7	-
8	6.37 (1H, d, 0.64)
9	-
10	-
1’	-
2’	8.12 (2H, m, 1.25)
3’	6.92 (2H, m, 1.26)
4’	-
5’	6.92 (2H, m, 1.26)
6’	8.12 (2H, m, 1.25)
1’’	5.34 (1H, dt, 0.65)
2’’	3.63 (6H, m, 4.71)
3’’	3.32 (2H, m, 1.07)
4’’	3.92 (1H, m, 1.43)
5’’	3.14 (1H, dtt, 1.07)
6’’	3.63 (6H, m, 4.71)
1’’’	4.54 (1H, m, 1.28)
2’’’	3.63 (6H, m, 4.71)
3’’’	3.63 (6H, m, 4.71)
4’’’	3.32 (2H, m, 1.07)
5’’’	3.63 (6H, m, 4.71)
6’’’	1.26 (3H, dd, 1.92)

^{1}H-NMR of Rutin

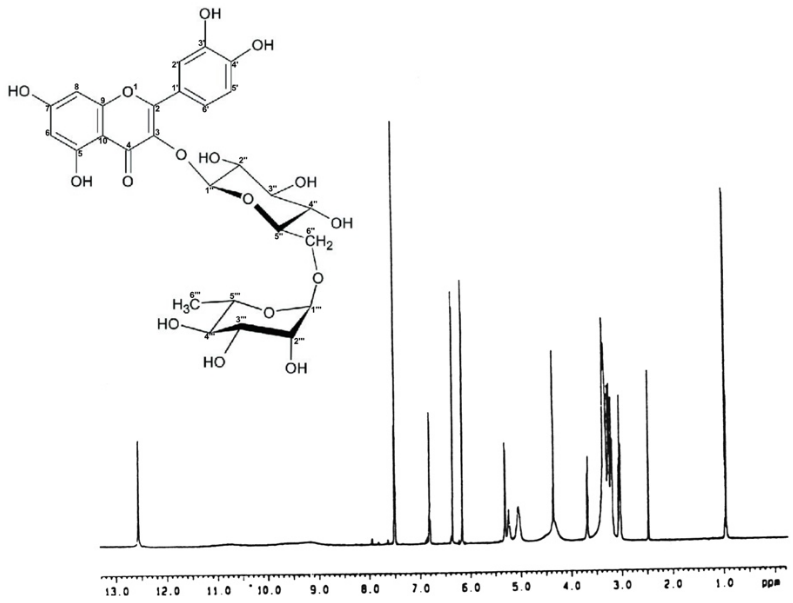

Position	Rutin<br>δH (int, mult., J (Hz))
1	-
2	-
3	-
4	-
5	-
6	6.37 (1H, d, 1.0)
7	-
8	6.21 (1H, d, 1.0)
9	-
10	-
1’	-
2’	7.62 (1H, m, 0.92)
3’	-
4’	-
5’	6.78 (1H, d, 0.48)
6’	7.50 (1H, dd, 0.82)
1’’	5.04 (1H, dt, 1.02)
2’’	3.63 (6H, m, 7.33)
3’’	3.32 (2H, m, 1.67)
4’’	3.42 (1H, m, 2.26)
5’’	2.48 (1H, dt, 0.98)
6’’	3.63 (6H, m, 7.33)
1’’’	4.44 (1H, m, 2.0)
2’’’	3.63 (6H, m, 7.33)
3’’’	3.63 (6H, m, 7.33)
4’’’	3.32 (2H, m, 1.67)
5’’’	3.63 (6H, m, 7.33)
6’’’	0.98 (3H, dd, 3.02)

^{13}C-NMR of Rutin Astragalin

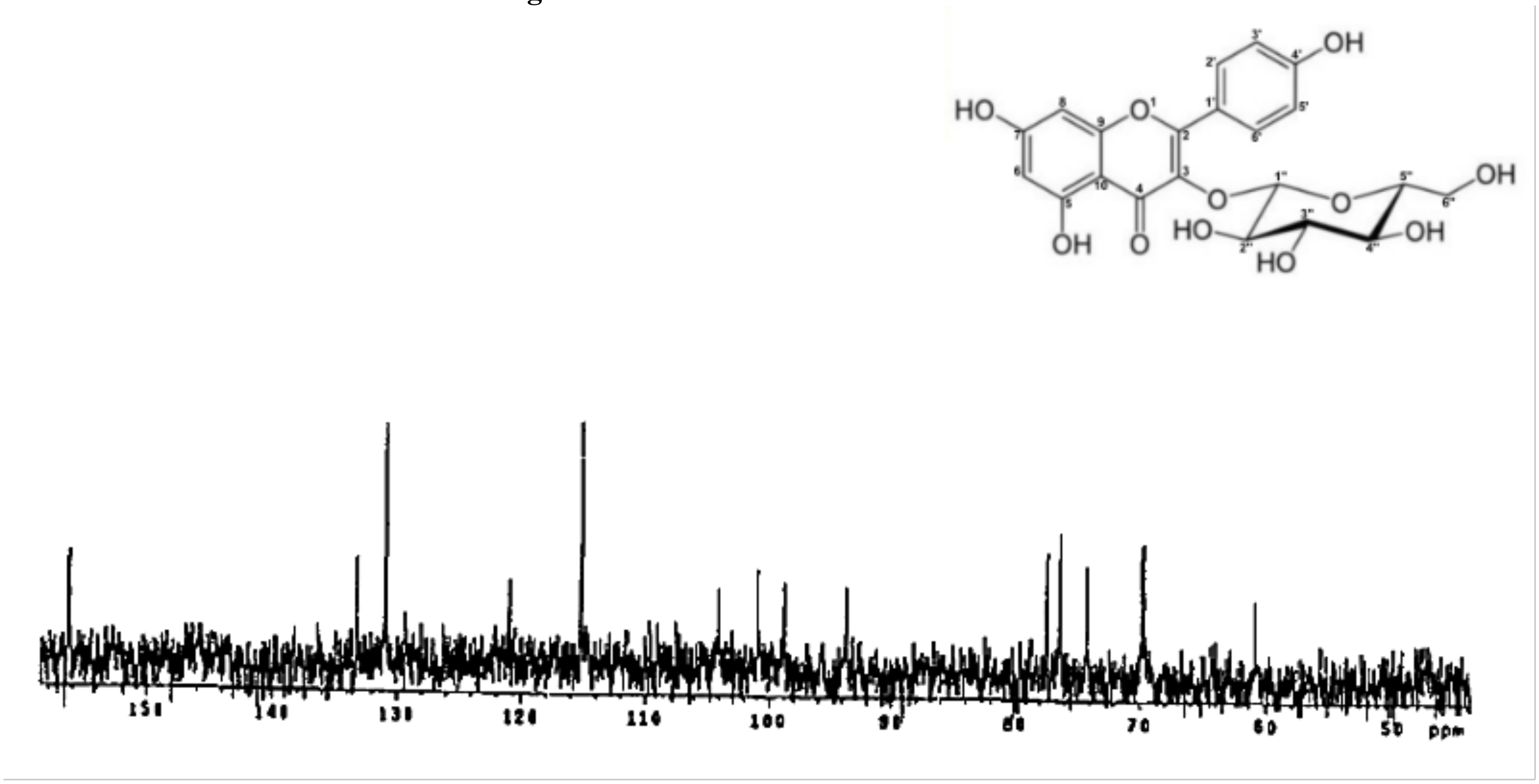

Position	Astragalin
	δC
1	-
2	142.82
3	133.65
4	155.50
5	141.54
6	99.73
7	145.88
8	94.85
9	149.22
10	105.67
1’	122.58
2’,6’	132.31
3’,5’	116.12
4’	135.47
1’’	103.87
2’’	75.68
3’’	77.94
4’’	71.31
5’’	78.41
6’’	62.64

^{13}C-NMR of Chlorogenic acid

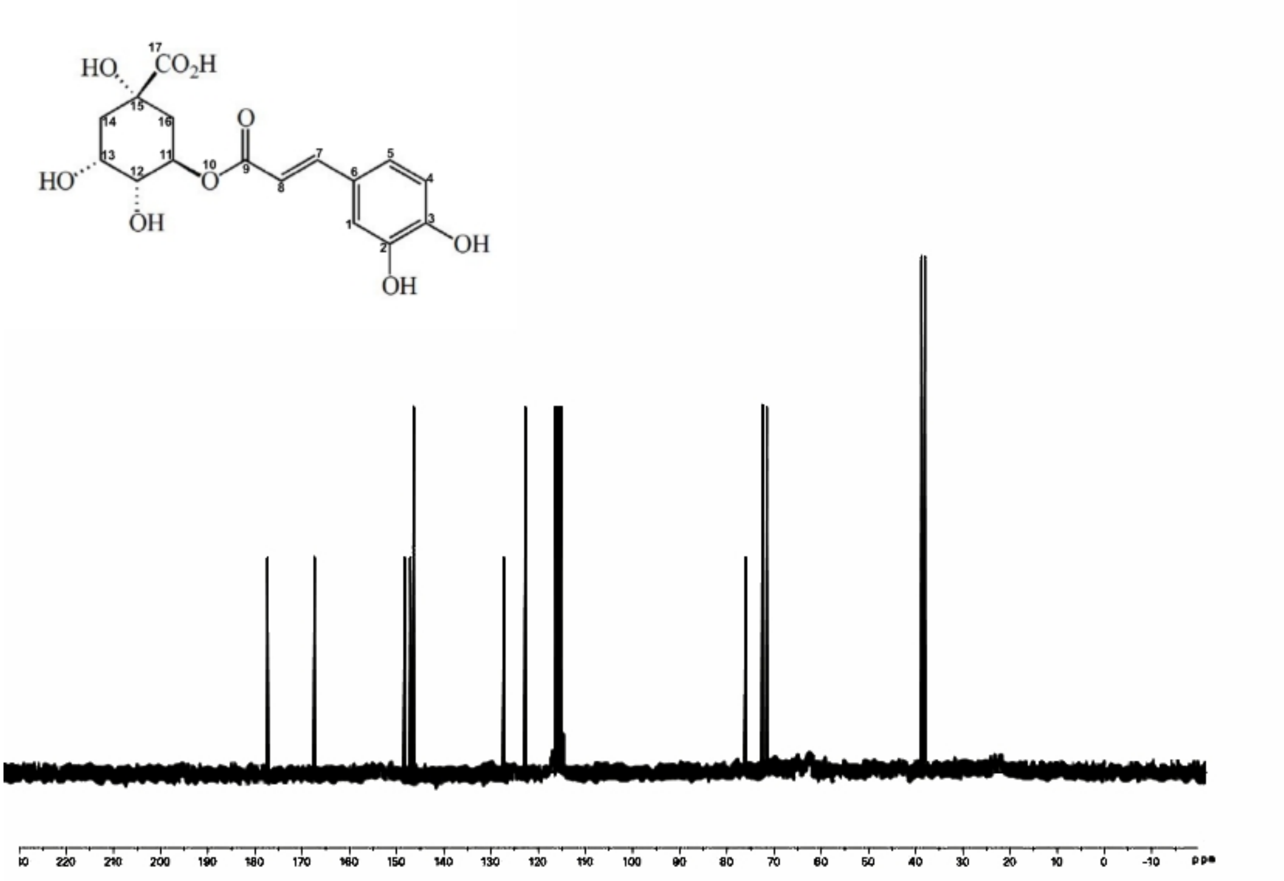

Position	Chlorogenic Acid
	δC
1	115.14
2	147.12
3	148.29
4	116.34
5	122.72
6	127.26
7	146.39
8	115.70
9	167.41
10	-
11	72.47
12	72.56
13	71.56
14	38.77
15	76.05
16	38.06
17	177.34

^{13}C-NMR of Hyperin

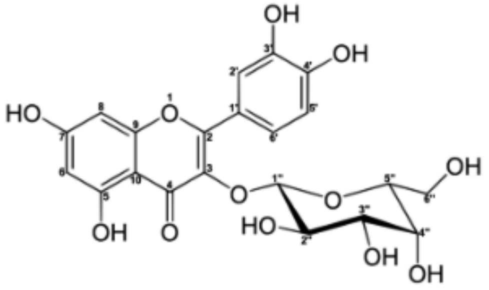

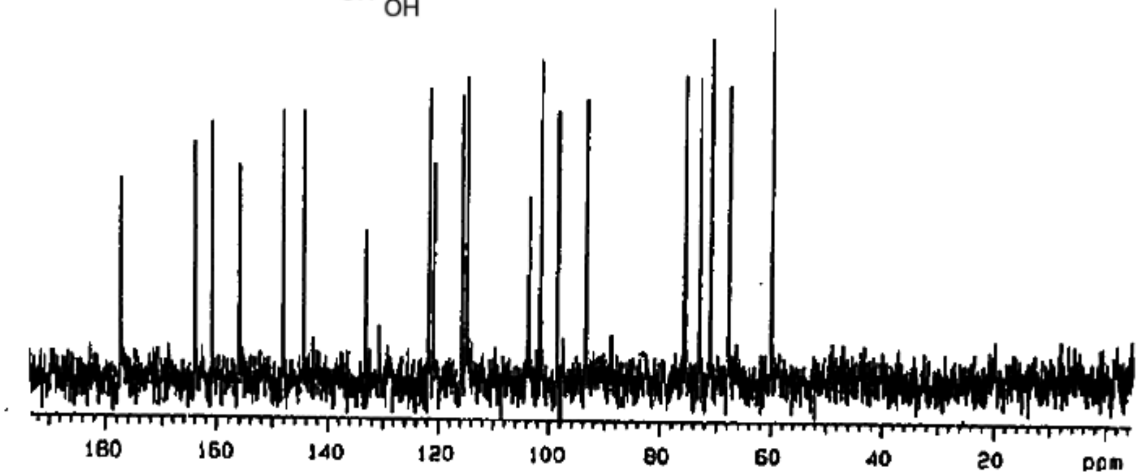

Position	Hyperin δC
1	-
2	157.32
3	136.00
4	178.26
5	161.84
6	99.25
7	165.02
8	94.34
9	157.47
10	104.79
1’	122.11
2’	116.34
3’	145.56
4’	148.78
5’	116.06
6’	122.29
1’’	102.85
2’’	72.06
3’’	74.01
4’’	69.67
5’’	76.35
6’’	59.61

^{13}C-NMR of Isoquercitrin

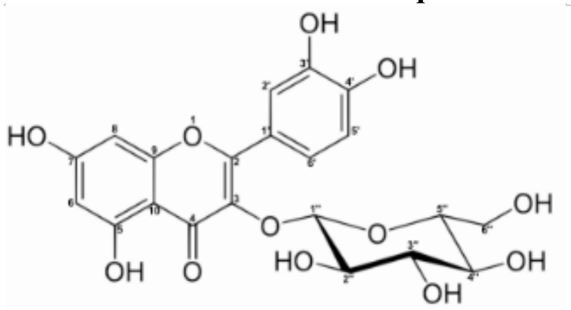

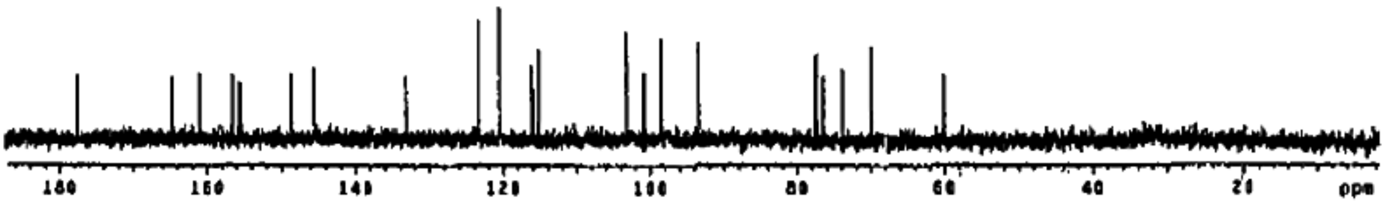

Position	Isoquercitrin
	δC
1	-
2	157.32
3	136.00
4	178.26
5	161.84
6	99.25
7	165.02
8	94.34
9	157.47
10	104.79
1’	122.11
2’	116.34
3’	145.56
4’	148.78
5’	116.06
6’	122.29
1’’	102.19
2’’	74.40
3’’	77.28
4’’	70.83
5’’	77.57
6’’	61.90

^{13}C-NMR of Myricetin

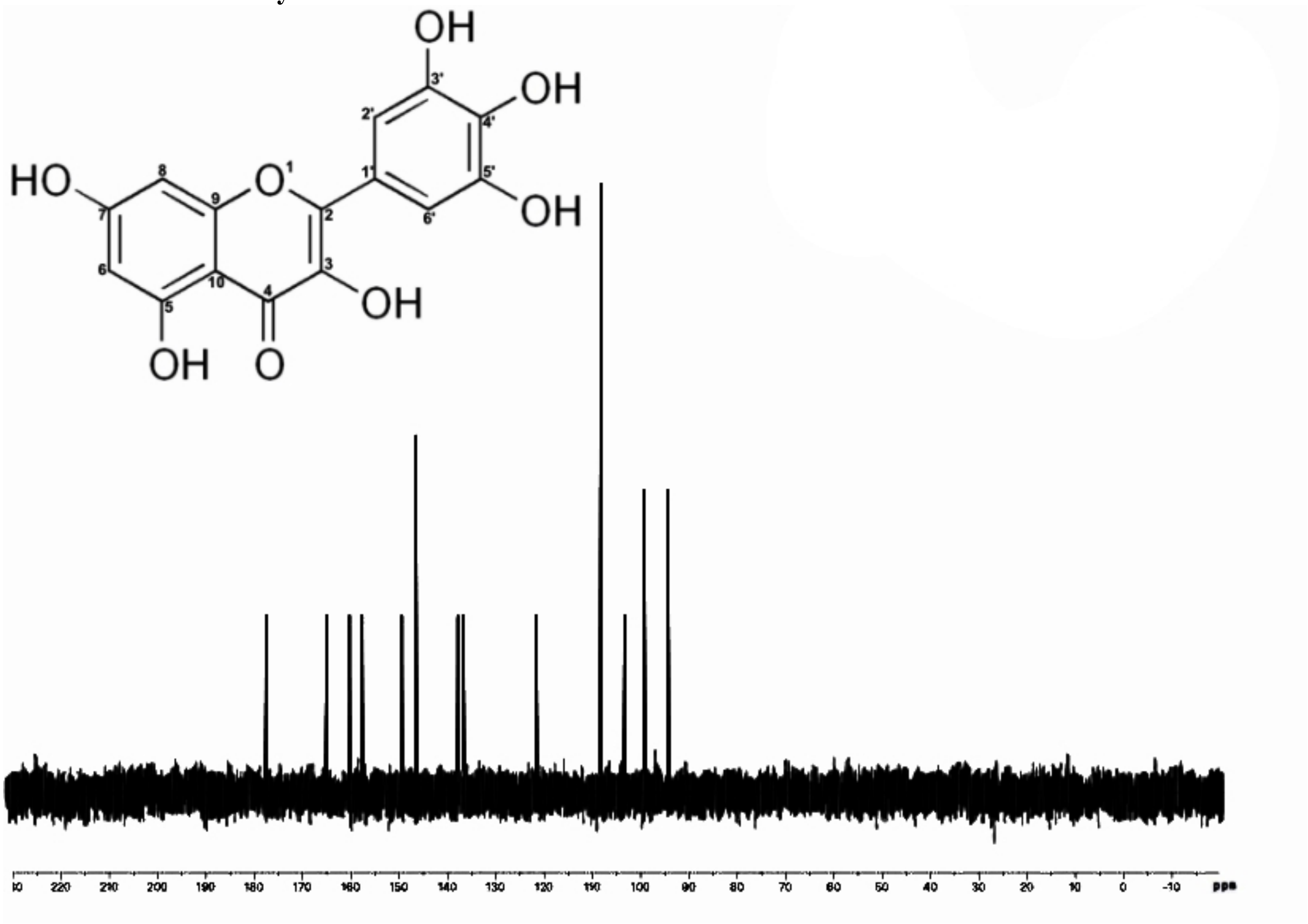

Position	Myricetin
	δC
1	-
2	149.41
3	136.67
4	177.52
5	160.23
6	99.25
7	165.02
8	94.34
9	157.64
10	103.38
1’	121.57
2’	108.23
3’	146.52
4’	137.88
5’	146.52
6’	108.23

^{13}C-NMR of Narcissin

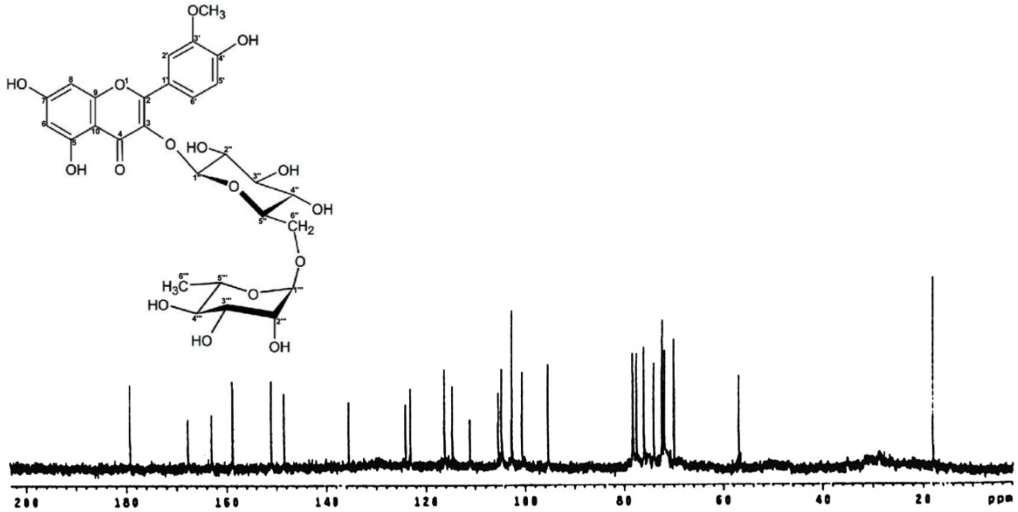

Position	Narcissin
	δC
1	-
2	157.38
3	134.13
4	178.26
5	161.84
6	99.25
7	165.02
8	94.34
9	157.47
10	104.79
1’	122.12
2’	114.24
3’	148.60
OCH_3	56.16
4’	149.01
5’	115.51
6’	122.77
1’’	102.58
2’’	74.70
3’’	76.82
4’’	70.88
5’’	76.65
6’’	67.28
1’’’	101.60
2’’’	71.61
3’’’	71.77
4’’’	73.30
5’’’	69.35
6’’’	18.10

^{13}C-NMR of Nicotiflroin

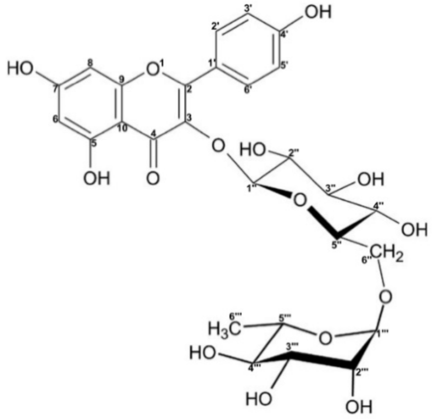

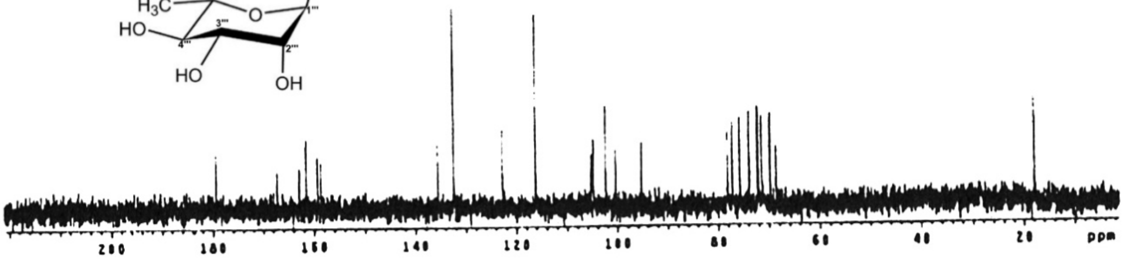

Position	Nicotiflorin
	δC
1	-
2	148.55
3	152.97
4	178.43
5	161.84
6	99.25
7	165.02
8	94.34
9	158.14
10	104.79
1’	121.62
2’	133.97
3’	115.79
4’	160.15
5’	115.79
6’	136.40
1’’	102.58
2’’	74.70
3’’	76.82
4’’	70.88
5’’	76.65
6’’	57.28
1’’’	101.60
2’’’	71.61
3’’’	71.77
4’’’	73.30
5’’’	69.35
6’’’	18.10

^{13}C-NMR of Rutin

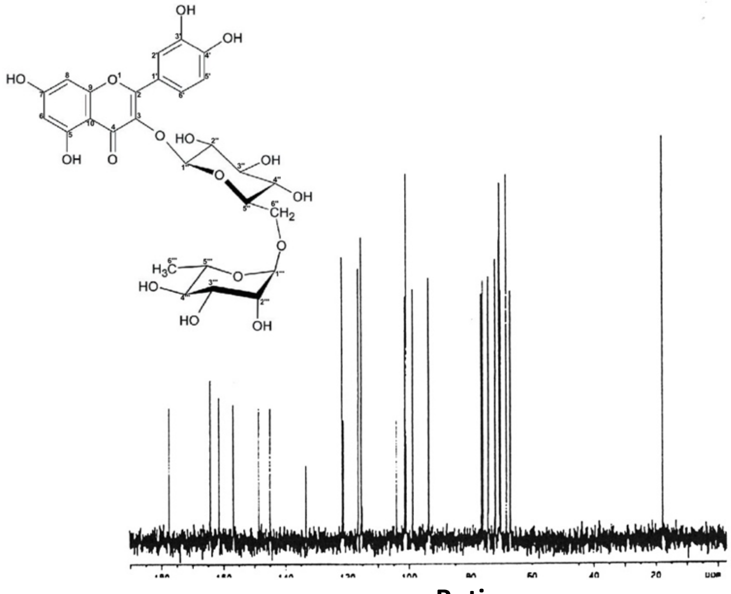

Position	Rutin
	δC
1	-
2	157.32
3	134.17
4	178.26
5	161.84
6	99.25
7	65.02
8	94.34
9	157.47
10	104.79
1’	122.11
2’	116.34
3’	145.56
4’	148.78
5’	116.06
6’	122.29
1’’	102.58
2’’	74.70
3’’	76.82
4’’	70.88
5’’	76.65
6’’	67.28
1’’’	101.60
2’’’	71.61
3’’’	71.77
4’’’	73.30
5’’’	69.35
6’’’	18.10
